# Embryonic timing, axial stem cells, chromatin dynamics, and the Hox clock

**DOI:** 10.1101/gad.303123.117

**Published:** 2017-07-15

**Authors:** Jacqueline Deschamps, Denis Duboule

**Affiliations:** 1 Hubrecht Institute, University Medical Center Utrecht, 3584 CT Utrecht, the Netherlands;; 2 School of Life Sciences, Ecole Polytechnique Fédérale, Lausanne, 1015 Lausanne, Switzerland;; 3 Department of Genetics and Evolution, University of Geneva, 1211 Geneva 4, Switzerland

**Keywords:** chromatin, collinearity, embryos, *Hox* genes, TAD, transcription, stem cells

## Abstract

Collinear regulation of *Hox* genes in space and time has been an outstanding question ever since the initial work of Ed Lewis in 1978. Here we discuss recent advances in our understanding of this phenomenon in relation to novel concepts associated with large-scale regulation and chromatin structure during the development of both axial and limb patterns. We further discuss how this sequential transcriptional activation marks embryonic stem cell-like axial progenitors in mammals and, consequently, how a temporal genetic system is further translated into spatial coordinates via the fate of these progenitors. In this context, we argue the benefit and necessity of implementing this unique mechanism as well as the difficulty in evolving an alternative strategy to deliver this critical positional information.

## Abstract (precis)

This review by Deschamps and Duboule discusses recent advances in collinear regulation of Hox genes in space and time as well as novel concepts associated with large-scale regulation and chromatin structure during the development of both axial and limb patterns.

“*In order to stop at any determined point in time*, *there is a lever to lock the triple brake*”

How to construct a time machine (Alfred Jarry 1899)

During the development of vertebrate animals, *Hox* genes provide positional information to the emerging embryonic axial tissues, thereby instructing them how to undergo appropriate morphogenesis (Krumlauf 1994; Wellik 2009). While this local patterning of embryonic tissues by various combinations of *Hox* proteins likely emerged early during evolution, the underlying molecular mechanisms are diverse, depending on the ontogenetic and phylogenetic contexts. The strong adaptive value of this system ultimately relied on distinct distributions of proteins rather than the genetic mechanisms used to deliver this information. Consequently, various mechanisms seem to have evolved in bilaterian animals, depending on their developmental strategies to secure an appropriate spatial coordination in *Hox* gene expression along the emerging embryonic axes. However, in many classes of bilateria, this coordination is associated with the process of “collinearity,” whereby clustered *Hox* genes are functional in a series of spatial domains that recapitulates the order of the respective genes in their genomic cluster (Lewis 1978; Gaunt et al. 1988). Nevertheless, this rule has many exceptions, and animals as diverse as the urochordate *Oikopleura* or the fruit fly *Drosophila* display collinear *Hox* gene expression either without any *Hox* clusters or with a split series of genes, respectively (Harding et al. 1985; Seo et al. 2004; see Duboule 2007), thus illustrating that various mechanisms can eventually deliver similar information.

Vertebrates organize their bodies through progressive growth from anterior to posterior structures. *Hox* genes are activated in a timed sequence, which follows their 3′-to-5′ genomic order—a process referred to as “temporal collinearity” (Dolle et al. 1989; Izpisua-Belmonte et al. 1991). This time-controlled transcriptional activation (the *Hox* clock) has been observed thus far only in animals containing an intact cluster of genes and occurs in a growth zone, a progenitor region located at the posterior aspect of the extending body axis. Therefore, temporal collinearity is a property displayed by animals with an anterior-to-posterior developmental progression in time, whereas strategies relying mostly on other developmental principles that do not involve a progressive anterior-to-posterior determination do not call for this process. As a consequence, they may not display an intact *Hox* cluster (Duboule 1994). In *Drosophila*, for instance, transcriptional activation of *Hox* genes is not sequential in time but instead depends on the regional activities of both gap and segmentation genes (Ingham and Martinez-Arias 1986; Casares and Sanchez-Herrero 1995).

Mammals achieve the appropriate *Hox*-mediated spatial patterning through the initial timed-sequenced activation of their *Hox* clusters in response to early embryonic signals (Forlani et al. 2003; Neijts et al. 2016). During axial elongation, they use this early timing mechanism to synchronize *Hox* gene expression with the progressive generation of the trunk and tail from the posterior embryonic growth zone (Neijts et al. 2016, 2017). This *Hox* clock is critical for the spatial distribution of the patterning information both in various axial tissues and along the appendicular axes (Fig. 1). Here we discuss recent progress in understanding the molecular mechanisms at work in mouse embryos, which orchestrate their *Hox* expression program during embryogenesis. In the context of a permissive genome topology, initial *Hox* gene activation responds to time-dependent embryonic signals acting on enhancers located on the early side of the cluster, and collinear *Hox* gene expression is relayed to the regional tissue anlagen that generates axial structures. The *cis*-acting mechanisms underlying this time-dependent developmental sequence may constitute an important constraint in the organization of an evolutionarily conserved Bauplan in vertebrates.

**Figure 1.**
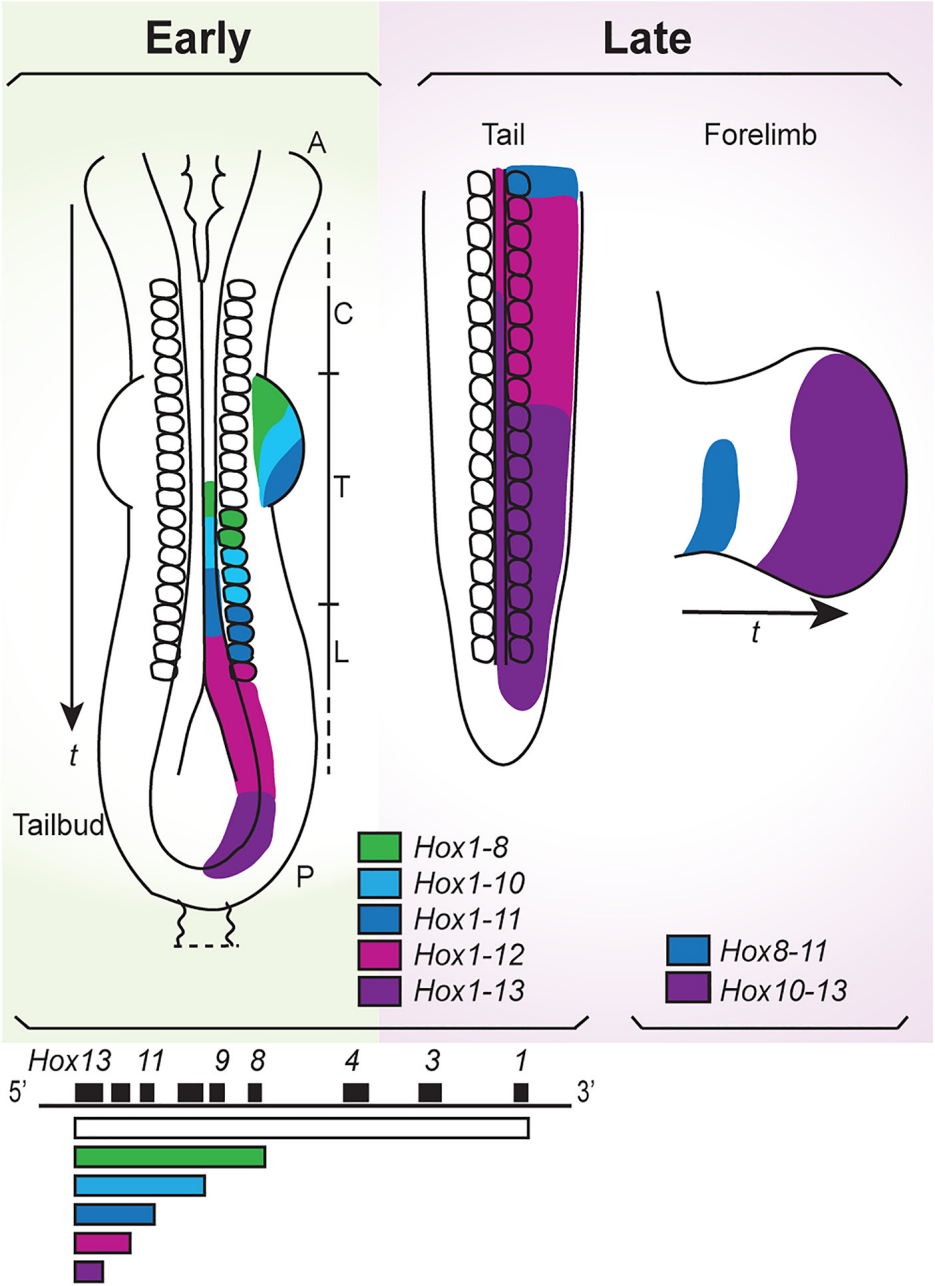
Collinear expression of *Hox* genes during the development of trunk axial tissues and limbs. (*Left*) Early: Schematic drawings of posteriorly overlapping transcript domains of *HoxD* genes in developing trunk axial tissues and early limb buds of an embryonic day 9.5 (E9.5) mouse embryo. *Hox*-expressing tissues are neural tube (midline); somites (blocks along the neural tube) labeled as cervical (C), thoracic (T) and lumbar (L); forelimb mesoderm (lateral bulges at the level of somites 7–12); and nascent mesoderm and neurectoderm in the tailbud. (*t*) Time of anterior to posterior development. Colinearity between the positions of the genes in the cluster and the anterior extension of their expression domains along the antero–posterior embryonic axis is illustrated both in the schematic embryo and by the bars *under* the cluster drawn *below*. *Hox1* is expressed from an anterior limit that is the most rostral of all *Hox* genes in the embryo (expression not shown in the embryo; no color in the bar), and *Hox8* is expressed from an anterior limit that is less rostral than that of *Hox1* in the embryo. Posterior to this *Hox8* anterior expression boundary, all genes between 1 and 8 are expressed (green color in the schematic embryo and in the bar corresponding to *Hox8*). Similarly, posterior to the *Hox10* expression boundary in the embryo, all genes between *Hox1* and *Hox10* are expressed (lighter blue in the schematic embryo and in the bar corresponding to *Hox10*), and similar representations illustrate the expression of *Hox11*, *Hox12*, and *Hox13*. These domains thus tend to overlap posteriorly in the embryo, like Russian dolls. Expression of other *Hox* genes is not shown. (*Right*) Late: *Hox* transcript distribution in the E10.5 developing tailbud and forelimb bud. The two domains in late forelimb buds mark the future proximal (arm and forearm) and distal (digits) parts of the adult limbs, respectively. (*t*) Time of development of proximal to distal limb structures. Color codes indicate the cumulative amounts of combinations of *Hox* transcripts. Anterior is to the top in all schemes.

## Embryonic timing and *Hox* gene expression

In murine embryos, the positional identities of post-occipital tissues (i.e., located from below the skull to more caudal areas) are acquired in progenitor cells located in the posterior embryonic growth zone. This zone includes the rostral third of the primitive streak as well as its flanking epiblast. Its posterior-most portion produces the lateral and paraxial mesoderm from mesoderm progenitors (MPs) (Lawson et al. 1991; Kinder et al. 1999), whereas the rostral-most aspect of the streak abutting the node (the node–streak border) contains stem cell-like progenitors for both the mesoderm and neurectoderm (Cambray and Wilson 2002, 2007). These latter bipotent progenitors, called neuromesodermal progenitors (NMPs), appear in the embryo at around embryonic day 7.5 (E7.5), just before the first somite is formed (Wymeersch et al. 2016). They constitute a tightly regulated and likely changing population of self-renewing cells, feeding the elongating axis with new mesoderm and neurectoderm tissues. As they differentiate, NMPs progressively populate more posterior embryonic axial tissues (Tzouanacou et al. 2009; Wilson et al. 2009). These progenitors, which are initially present in the anterior streak, are subsequently found in the tailbud between E10.5 and E13.5, where they contribute to the formation of the most caudal part of the axis.

**Figure 2.**
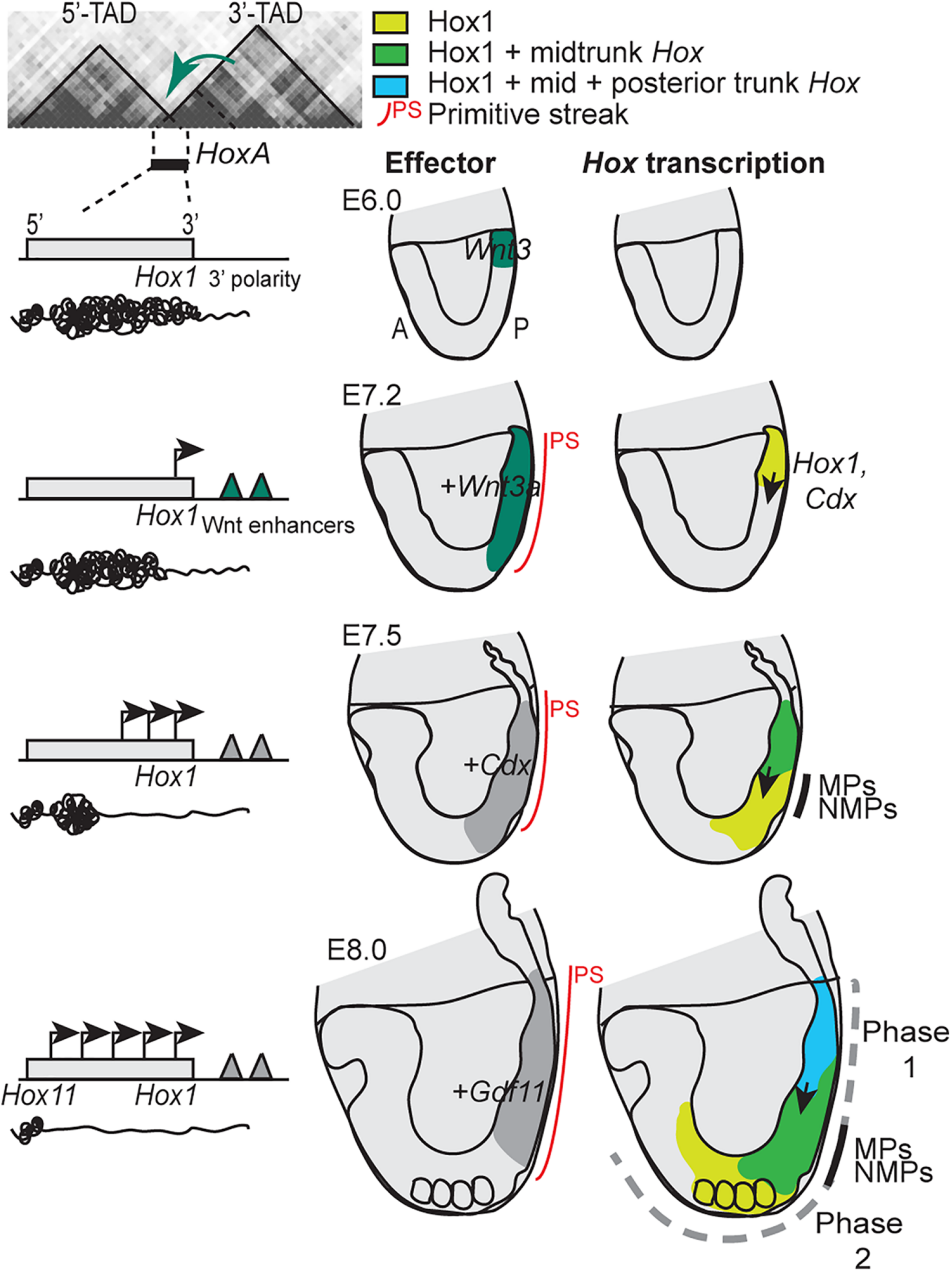
Temporal collinearity of *Hox* gene transcription is relayed by axial progenitors into spatially collinear expression domains. Schematics showing the early expression of *HoxA* genes. The *HoxA* cluster is represented in reversed genomic orientation in order to appear comparable with *HoxD* (Fig. 3). Out of the *Hox1* to *Hox13* genes, only the 3′-most-located *Hox1* and 5′-most-located gene expressed at the stages considered (*Hox11*) are indicated *below* the cluster (light-gray box). (From *top* to *bottom*) Developmental stages and associated genomic events at the *Hox* locus (*left*) and transcription domains of *Hox* genes (embryos at the *right*) and of their inducers (embryos on the *left*). (E6.0) Preferential interactions between 3′-located *Hox* genes and the 3′ topologically associating domain (TAD). No *Hox* expression was detected, but priming of the 3′ flanking region (open chromatin) is shown in the *Wnt3-*expressing region. (E7.2) *Hox* (and *Cdx*) transcriptional initiation by *Wnt3* and *Wnt3a* via *Wnt*-dependent enhancers in the proximal 3′ flanking region. (Light green) Expression domain of *Hox1* and *Cdx*. (E7.5) Mid-trunk *Hox* gene activation (dark-green domain) through the binding of *Cdx* products in the cluster and further chromatin opening. Mid-trunk *Hox* domains reach the axial progenitor region (or the posterior growth zone) in the anterior part of the streak. (E8.0; early somite) *Gdf11-*induced activation of posterior trunk *Hox* genes (blue). Expansion of trunk *Hox* gene transcripts (*Hox1* [light green] and mid-trunk *Hox* [dark green]) into the axial progenitor region (end of phase 1). The color code indicates cumulative amounts of *Hox* transcript combinations. The expression domains overlap posteriorly, as explained in the legend for Figure 1. For all embryos, anterior is to the left. (PS) Primitive streak. (Arrows) transcriptional activation. Genome topology at the *top left* is from Dixon et al. (2012).

*Hox* genes are initially switched on in the posterior streak area as a response to *Wnt3* signals (Neijts et al. 2016). Their expression domains subsequently spread anteriorly through a process that does not involve cell migration (Deschamps and Wijgerde 1993; Gaunt and Strachan 1994). The rostral expansion of the *Wnt3* (and later *Wnt3a*) domain at gastrulation is followed by a similar expansion of 3′-located *Hox* transcripts toward the progenitor region (Fig. 2). Successively more 5′-located *Hox* genes are induced thereafter and follow the same dynamics. As a result, the anterior streak region, which contains the MPs and NMPs, sequentially transcribes an increasing number of more “posterior” *Hox* genes. Thus, an early *Wnt*-dependent wave of collinear *Hox* gene transcription along the streak (phase 1 expression) instructs MPs and NMPs, the descendants of which assume embryonic spatial patterning. In this context, early to later axial progenitors acquire group 1–13 combinatorial *Hox* addresses at the time that they release descendants that generate tissues at various future anterior to posterior body levels. This process provides synchronicity between the acquisition of genetic positional information over time (temporal collinearity) and the anterior to posterior levels where progenitors carrying this information contribute during axial elongation (spatial collinearity)—a translation from temporal information to a set of spatial coordinates.

## Directionality of the *Hox* clock

In vertebrates*,* all *Hox* genes of a cluster are transcribed from the same DNA strand, and the first to be expressed are members of groups 1 and 2 (i.e., those genes located at “3′ ends” of the gene clusters) followed by more 5′-located genes. However, while an inversion in this transcriptional directionality (starting from group 13) led to early lethality (Zakany et al. 2004), the factors determining and initiating this obligatory time sequence are not yet fully understood. They may involve a pre-existing favorable genomic topology, which would skew gene activation toward one side of the gene clusters. The asymmetric distribution of *Wnt*-responsive enhancer sequences on the early side of the cluster (Fig. 2; Neijts et al. 2016) also likely constitutes such a bias. A few hours after, the more centrally located *Hox* genes belonging, for example, to groups 4–10 are activated, and their transcription is further enhanced by a feed-forward effect of *Cdx* gene products, which are *Wnt*-dependent transcription factors initially expressed like 3′-located *Hox* genes. This stimulating effect of *Cdx* proteins over these central *Hox* gene transcriptions (Neijts et al. 2017; see also Bel-Vialar et al. 2002; van den Akker et al. 2002) seems to occur through binding sites within the *Hox* clusters, as shown in the case of the *HoxA* locus (Mazzoni et al. 2013; Amin et al. 2016; Neijts et al. 2017).

On the other hand, enhancement of transcription of the more 5′-located “posterior” *Hox* genes (e.g., group 11) responds to the presence of *Gdf11*, a TGFβ signal released by posterior embryonic tissues after E8.0 (Liu et al. 2001; Liu 2006; Jurberg et al. 2013). Therefore, beyond a pre-existing chromatin architecture, the precise timing of distinct signals during the progression of embryogenesis appears to determine a global collinear activation of *Hox* gene transcription: Wnt3 and Wnt3a signaling induces anterior genes (Neijts et al. 2016), and Cdx proteins stimulate trunk/central genes (Neijts et al. 2017), whereas Gdf11 further activates group 11 *Hox* genes (Gaunt et al. 2013) as well as groups 12 and 13 (our unpublished data). While the temporal progression within these various groups of genes responding to the same factor remains to be understood, it may rely on a progressive and directional opening of a chromatin structure (see below).

## Axial progenitors translate the *Hox* clock into spatial coordinates

The onset of temporal collinearity is determined by *Wnt* signaling, a parameter dependent on the developmental timing of the embryo itself (Neijts et al. 2016). In contrast, the end of sequential *Hox* gene activation is fixed by the transcription of the last genes of the clusters, members of group 13 (Neijts et al. 2017). These genes play a crucial role in retroinhibiting the functions of more anterior HOX proteins and arrest axial elongation (Young et al. 2009), thus explaining the early lethality of mice carrying an inverted *Hox* cluster (see above). This negative feedback may involve a dominant-negative effect, referred to as posterior prevalence; i.e., the prevalent function of posterior HOX proteins over their more anterior neighbors. This effect was observed in many instances and may rely on various mechanisms (e.g., see Gonzalez-Reyes et al. 1990; Duboule and Morata 1994; Yekta et al. 2008; Durston and Zhu 2015). It coincides with both the slowing down of axial tissue production and a reduction in the size of the presomitic mesoderm (PSM) (Gomez and Pourquie 2009; Denans et al. 2015). In this view, the *Hox* clock must be tightly synchronized with the intrinsic timing of the embryo, the developmental clock.

**Figure 3.**
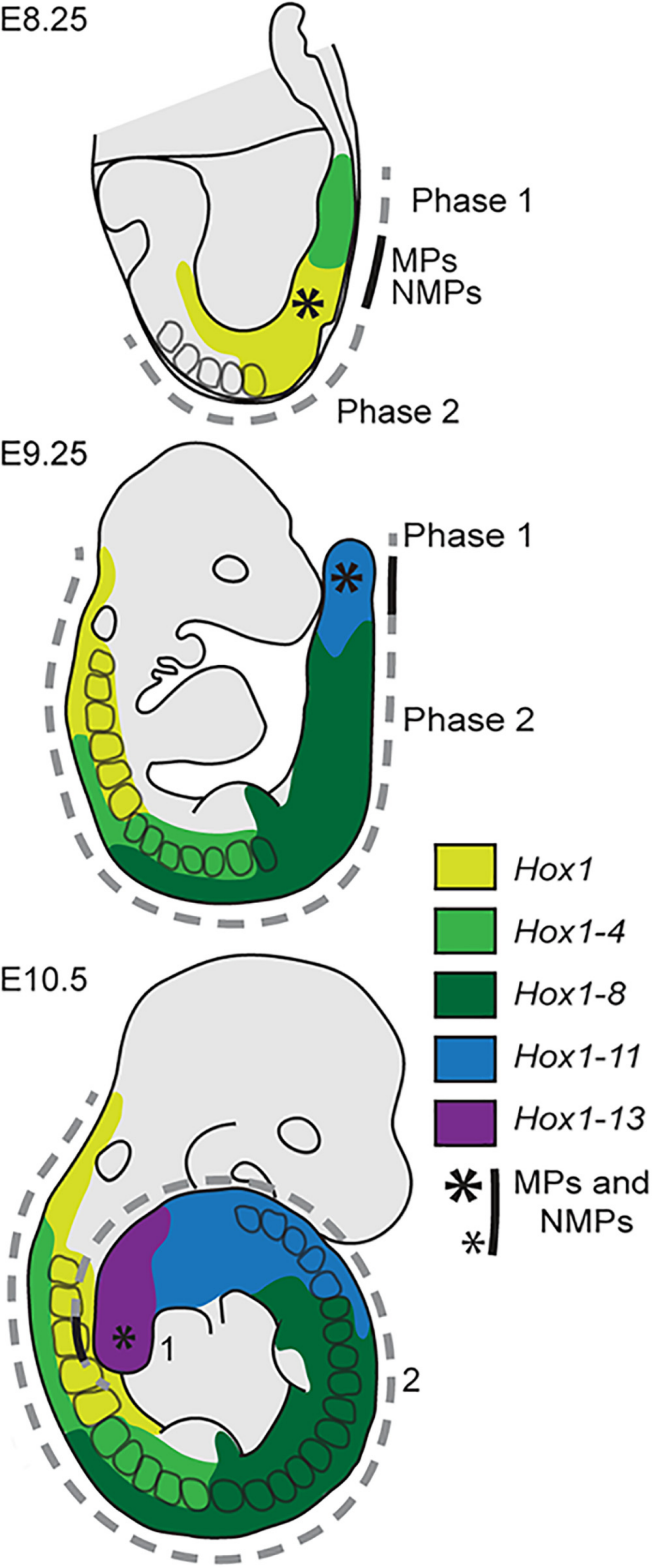
Early temporal collinearity of *Hox* gene transcription is relayed by the axial progenitors into spatially collinear expression domains in the emerging differentiated tissues. (E8.25) The transcript domains of *Hox1* followed by that of *Hox2* to *Hox4* (phase 1, temporal collinearity) expand anteriorly and reach the axial progenitor region ([*] MPs and NMPs) before expanding further anteriorly during phase 2. (E9.25) Transcriptional initiation of the next *Hox* genes has occurred (mid-trunk *Hox4* to *Hox8*), and activation of more posterior *Hox* genes takes place (as shown with *Hox11*). The expression domains expand anteriorly toward the axial progenitor area in those tissues generated by the descendants of the MPs and NMPs (phase 2, spatial collinearity). (E10.5) *Hox13* is now transcriptionally activated and expressed strongly posteriorly, where it counteracts further axial extension. The *Wnt* and *Fgf* pathways are weakened, and the progenitor niche becomes deficient, with fewer and fewer MPs and NMPs (smaller asterisk). The color code identifies the combinations of *Hox* genes expressed along the axis at the different stages. MPs and NMPs are found around the anterior part of the primitive streak, just posterior to the node (indentation) at E8.25 and in the tailbud at E9.25 and E10.5. For the E8.25 embryo, anterior is to the left; for the E9.25 and E10.5 embryos, anterior is to the top.

Because these initial collinear transcript domains expand anteriorly into the region of axial progenitors, early MPs and NMPs express 3′-located *Hox* genes exclusively and thus have an anterior identity, whereas progenitors present at a later time express more 5′-located *Hox* genes in addition to anterior genes and thus have a more posterior identity (Figs. 2, 3). Subsequently, progenitors transmit these identities to their differentiated cell progenies, which contribute to the elongating tissues along the growing axis. The transmission of these identities marks the end of the early phase of *Hox* gene transcription (phase 1) (Figs. 2, 3). A second phase of *Hox* expression (phase 2) then takes place as the axial progenitors generate the elongating neurectoderm and mesoderm, with different regulatory processes depending on both the germ layer and the axial level.

Therefore, *Hox* patterns are established in time in a collinear manner during phase 1 and instruct early to late axial progenitors about their anterior to posterior identity, respectively. These identities are further transmitted to their differentiated progenies, which expand and contribute to axial extension. These *Hox* patterns are maintained during phase 2, which occurs at early somite stages for 3′-located (or “anterior”) *Hox* genes (Figs. 2, 3) and continues until after the embryo has turned, when progenitors for the posterior trunk are located in the tailbud (Fig. 3, E9.25 embryo). This lasts until E10.5, when the tailbud contains only a decreasing population of progenitors for tail tissues. Therefore, from an early to a late developmental stage, axial progenitors for anterior to more posterior axial tissues first acquire their *Hox* addresses and subsequently relay this information to their progeny in the emerging differentiated neurectoderm and mesoderm.

On top of these global regulations, additional gene- and/or tissue-specific regulatory events may fine-tune the *Hox* expression domains in axial tissues. While the rostral expression boundary of any given *Hox* gene will eventually be more anterior in the neurectoderm than in the mesoderm, which may reflect distinct properties of these cellular subsets produced by the progenitor pools, expression might either be reinforced by autoregulatory loops (Popperl et al. 1995) or undergo a late retinoic acid-dependent anterior shift in other cases (Oosterveen et al. 2003).

## Relay from early temporal to spatial *Hox* expression: a mechanism conserved in vertebrates

Recent findings in zebrafish (e.g., Steventon et al. 2016) in addition to previous literature suggest that the translation of an initial *Hox* expression timing into successive anterior-to-posterior instructions to axial tissues is a feature conserved throughout vertebrates despite the apparent differences in how these organisms gastrulate. In amphibian and murine embryos, for example, *Wnt* signals precede the onset of *Hox* gene activation in a ring around the marginal zone in *Xenopus laevis* (Holland 2002) or along the corresponding structure in mice—the primitive streak, respectively. In both species, sequential *Hox* gene transcription further expands into presumptive trunk tissues as gastrulation proceeds, as schematized for mice (Figs. 2, 3; see also Deschamps and van Nes 2005) and described for *Xenopus* (Wacker et al. 2004). However, slight differences exist; *Hox* transcripts are found in a large marginal zone in amphibians that converges toward the blastopore lip, whereas the tiny initial murine *Hox* domains expand anteriorly along the streak toward the regions containing MPs and NMPs. The situation in bird embryos is somewhat similar to that in mice (Iimura et al. 2007; McGrew et al. 2008), while the situation in fish is closer to that of amphibians (Alexandre et al. 1996). The embryonic domain fated to contribute to trunk tissues is thus larger in early fish and amphibian embryos than in mice, where a small population of axial progenitors ensures most of the axial growth in the trunk. In zebrafish, NMPs contribute significantly to axial growth only in the tailbud (Martin and Kimelman 2008; Steventon et al. 2016), and a low number of multipotent progenitors are found at relatively late stages in the *Xenopus* tailbud (Davis and Kirschner 2000).

Despite these differences, the translation of sequential *Hox* gene activation into spatial cues in axial tissue anlagen occurs in all vertebrates thus far examined, including amphibians (Durston et al. 2012). Since this feature is crucial for patterning along the main axis and because it is dependent on a timing device that is itself intrinsically linked to the physical arrangement of genes along a genomic cluster, we conclude that the meta-*cis* arrangement of *Hox* genes is constrained by the necessity to properly and safely implement the *Hox* clock. The existence of this particular constraint in all vertebrates was proposed earlier to coincide with the narrow passage of the phylotypic hourglass; i.e., a short time window during which various developmental strategies converge toward completion of axial growth (Duboule 1994; Raff 1996). As we discussed here, recent data indicate that the core of this obligatory process includes the relay of temporally acquired *Hox* codes to the precursors of anterior to posterior axial tissues.

## A link between the *Hox* clock and the somitic clock?

Once the successive waves of *Hox* gene expression have progressively labeled the axial progenitors for trunk tissues in the node-abutting posterior growth zone (Figs. 2, 3), the progenitors transmit their *Hox* addresses to their daughter cells in both the mesoderm and neurectoderm of the emerging axial tissues. Early progenitors relay an anterior code, while later progenitors transmit a more posterior genetic address until the progenitor population is exhausted by the end of axial elongation. In nascent paraxial mesoderm, the proliferation of *Hox*-instructed cells takes place concomitantly with the segmentation of the PSM into somites, a series of discrete compartments that foreshadows the future vertebrae. The sequential production of somites results from an oscillatory mechanism (the “somitic clock”) (Palmeirim et al. 1997), and potential connections between the two clocks were documented in both mice (Zakany et al. 2001) and chicks (Dubrulle et al. 2001), where early expression of several anterior *Hox* genes was observed to cycle and follow a particular phase of the somitogenesis cycle (Pourquie 2003 and references therein).

Nevertheless, this connection between both clocks has not yet been functionally validated, and thus a mechanism synchronizing these clocks is still elusive. The necessary genetic approaches are complicated due to the high level of redundancy found in the developing PSM, where all four *Hox* gene clusters are at work. Alternatively, the somitic clock may operate independently from the *Hox* clock; i.e., by segmenting a tissue where the set of *Hox* addresses would already be properly distributed due to its earlier activation.

## *Hox* genes and the control of body length

The length of the trunk depends on the activity of axial progenitors. In addition to niche factors, which are essential for maintaining these progenitors (Wilson et al. 2009; Neijts et al. 2014), the pluripotency factor Oct4 is a crucial player in determining progenitor activity. A sufficiently high level of *Oct4* expression in the posterior aspect of the early mouse embryo is essential to maintain the pluripotency network active in the epiblast, and *Oct4* levels normally decrease at the three- to five-somite stage (Osorno et al. 2012). Experimental stimulation of early *Oct4* expression produced a longer trunk in mice, as shown by using the *Cdx2* promoter driving *Oct4* in early mouse embryos. Aires et al. (2016) increased both the level and the time of expression of *Oct4* in posterior epiblasts, including in the caudal lateral epiblast and the NMP region. This presumably overruled the reduction in *Oct4* expression and associated decline in pluripotency, which normally occurs in posterior epiblasts after the three- to five-somite stage, and allowed for an extended activity of the pluripotency network, leading to a longer trunk.

*Hox* genes, together with the related *Cdx* genes, also play a role in trunk extension. Therefore, they do more than merely confer axial identity to the emerging tissues. *Cdx* genes are required in a dosage-dependent manner for the generation of post-occipital embryonic axial tissues (van Rooijen et al. 2012). *Cdx2* transcriptionally activates *Wnt* and *Fgf* signaling pathway components in posterior embryonic tissues (Amin et al. 2016) and thus maintains the proficiency of the axial progenitor niche. Accordingly, experimental stimulation of both the *Wnt* and *Fgf* pathways rescues the axial truncation of *Cdx* mutants at least in part (Young et al. 2009; van Rooijen et al. 2012). Middle or trunk *Hox* genes can substitute for *Cdx* genes in axial extension, demonstrating that these *Hox* gene products stimulate trunk growth, presumably by maintaining a proficient niche of axial progenitors. The trunk growth-stimulating action of *Cdx* and *Hox* genes is amplified by a feed-forward activation of *Hox* genes by *Cdx2* (Neijts et al. 2017).

After the trunk-to-tail transition, posterior *Hox* genes become highly expressed in most caudal embryonic tissues. These genes—in particular *Hox13*—retroinhibit more anterior *Hox* genes (Denans et al. 2015; Beccari et al. 2016; Sheth et al. 2016). In addition, they antagonize *Cdx2* and more centrally located *Hox* genes in their task of axial stimulation by directly interfering with activation of the Wnt and Fgf pathways (Amin et al. 2016). Posterior *Hox* genes thus intervene to slow down and interrupt the axial elongation process by preventing *Cdx* and middle *Hox* genes from further activating axial progenitors, MPs, and NMPs at caudal axial levels.

## Early temporal collinearity is required for subsequent expression during development

The time-sequenced activation of murine *Hox* genes in the posterior primitive streak (Fig. 2, phase 1) inherently leads to their sequential transcription in the precursors of axial tissues. This corollary is also observed in birds (Iimura and Pourquie 2006), amphibians (Wacker et al. 2004), and fish (Alexandre et al. 1996), indicating that this feature is an evolutionarily conserved mechanism, fundamental for the organization of the vertebrate body plan. Here again, the underlying molecular mechanism is not yet fully understood. It nevertheless involves the concomitant and directional release of H3K27me3 (Soshnikova and Duboule 2009; Neijts et al. 2016), a histone H3 modification introduced by the *Polycomb*-repressive complex 2 (PRC2) and specific for inactive chromatin regions (see Margueron and Reinberg 2011). Through the recruitment of PRC1, these negative domains form compact structures from which transcribed *Hox* genes selectively escape, depending on the developmental context (Noordermeer et al. 2011; Mazzoni et al. 2013; Kundu et al. 2017).

At developmental time points earlier than the gastrulation stage epiblast, as in inner cell mass (ICM)-derived embryonic stem cells (ESCs), *Hox* genes are not transcribed. In these cells, the PRC2 coverage is not as dense as in *Hox*-negative differentiated cells (Noordermeer et al. 2011), likely due to the presence of bivalent positive and negative chromatin modifications of histone H3 (Bernstein et al. 2006). This moderately compacted poised state can also be observed through high-resolution stochastic optical reconstruction microscopy (STORM) (Fabre et al. 2015). These observations suggest that *Hox* gene expression and function at later developmental stages and in adult tissues are contingent on their initial phase of activation, which thus fixes the part of the gene cluster that remains at work at a given body level (the “open for business” model) (Duboule 1992 and references therein; Gaunt 2015 and references therein; Maeda and Karch 2015 and references therein).

## Open for business or open by business?

While the mechanism underlying the “open for business” model comprises similar components in flies and mammals (i.e., the establishment of dynamic “open” and “closed” chromatin domains early on) (Soshnikova and Duboule 2009; Bowman et al. 2014), the mammalian version implies that the extent of such domains is regulated initially as a function of time, unlike in *Drosophila*. In fact, the highly versatile removal of PRC-dependent histone modifications on chromatin along with the progression of transcription in time and the concurrent change in the three-dimensional (3D) structure (Noordermeer et al. 2014) raise the question of causality: Do chromatin domains set or maintain the expression of subsets of *Hox* genes or, alternatively, only reflect the transcriptional mechanisms at work?

While recent work in *Drosophila* suggests a causal role for *Polycomb*-dependent chromatin modifications in the epigenetic memory of a given transcriptional state (Coleman and Struhl 2017), the situation in vertebrates, which lack clear *Polycomb* response elements (PREs), is unclear. The cellular memory of various combinations of transcribed *Hox* genes and hence their transmission to daughter cells may indeed rely on a reinforcement of PRC-based compaction of the silent parts of *Hox* clusters after an initial temporal activation in early embryos. However, this activation may not depend on the removal of “negative” chromatin marks but instead may trigger this transition in chromatin structure. *Hox* genes might thus remain differentially active in the progenies of axial stem cells (Fig. 2, phase 2) either permanently or transiently. Interestingly, silent genes may be reactivated at later time points in various cells and tissues where their function was subsequently co-opted during evolution. In such cases, subgroups of *Hox* genes are often found (re)activated together, as in the cases of spinal motoneurons (Dasen and Jessell 2009; Mazzoni et al. 2013), the intestinal caecum (Delpretti et al. 2013), or the developing metanephros (Di-Poi et al. 2007), in response to potent remote enhancer sequences.

The various subgroups of contiguous *Hox* genes showing either sustained transcription or subsequent reactivation could be defined by the distribution of bound CTCF proteins, as the deletion of specific CTCF-binding sites within the *HoxA* and *HoxC* clusters was found recently to locally disturb the organization of chromatin domains and cause *Hox* gene misregulation, leading to patterning defects and motoneuron misspecification in mice (Narendra et al. 2015, 2016). As active versus inactive domains of *Hox* clusters are labeled by the absence or presence of *Polycomb* complexes, an interplay between these complexes and CTCF to establish these domains may occur (Narendra et al. 2015). In some normal or pathological cases, however, particular *Hox* genes seem to be transcribed in isolation from their immediate neighbors (e.g., see Seifert et al. 2015), thus escaping any type of collinear regulation, a situation difficult to reconcile with the above-mentioned hypothesis.

## Genomic topologies and dynamic architectures

The collinear regulation observed during trunk extension (described above) has long been thought to rely mostly on mechanisms involving regulatory sequences internal to the gene clusters (Spitz et al. 2005), such as enhancer sharing (Gould et al. 1997), based on results from transgenic animals where isolated (groups of) *Hox* genes were activated at about the right anterior–posterior positions. However, recent work indicates that the surrounding 3D chromatin architecture plays a critical role. Indeed, both the correct implementation of the collinear mechanism and the selection of which subgroups of *Hox* genes ought to be transcribed at any given time in various embryonic and adult contexts as well as in stem cells depends at least in part on regulatory sequences located outside the gene clusters.

Both *HoxA* and *HoxD* (i.e., the two mammalian clusters showing the most compact and complete structure) are flanked by two topologically associating domains (TADs), which are constitutive chromatin domains (Dixon et al. 2012; Nora et al. 2012) containing distinct sets of enhancers (Andrey et al. 2013; Berlivet et al. 2013; Neijts et al. 2016). At the *HoxA* locus, the 3′-located TAD (referred to here as “3′ TAD”) appears to contain enhancers necessary for activation of the earliest *Hox* gene (Fig. 2; Neijts et al. 2016; Cao et al. 2017), as if interactions between the gene cluster and the flanking regulatory landscape were required to properly control the timing of the system. Deletion of two discrete 3′ enhancers was shown to impair *HoxA* activation and strongly alter the profile of 3D interactions between *Hoxa1* and its 3′ and 5′ surroundings in differentiating ESCs (Cao et al. 2017). This shows that 3D chromatin architecture at the 3′ side of the *Hox* locus is dynamic and intimately linked to enhancer-driven gene activation. This may be one of the reasons why global interactions between *Hox* genes and this particular 3′-located landscape seem to be evolutionarily more conserved than interaction occurring at the 5′ side of the gene cluster, as shown by the presence of a single large TAD in *amphioxus* (Acemel et al. 2016), whereas the presence of two distinct TADs was observed from fish to mammals (Woltering et al. 2014). Subsequently, these two TADs help to partition regulatory inputs to those subsets of *Hox* gene targets that are transcribed in a variety of contexts. This segregation of productive interactions between either 3′-located *Hox* genes and their associated 3′ TAD or 5′-located genes and the other TADs have been well characterized in the context of appendicular development.

## Limb axes (temporal collinearity)^2^

Once progressive *Hox* gene activation has occurred, driven by sequences within the 3′ TAD, different parts of the *Hox* cluster are recruited along with the development of specific structures but always in relation to either one of the two flanking TADs. For example, the subset of *Hoxd* genes transcribed in the developing caecum or in mammary buds interacts only with the 3′ TAD, whereas another subset of genes expressed in the developing genitalia is regulated by sequences located within the 5′ TAD (Delpretti et al. 2013; Lonfat et al. 2014; Schep et al. 2016). This selectivity and exclusiveness in TAD functions (the 5′ TAD and 3′ TAD are never active at the same time) underlie collinear regulation during the development of our appendicular axes. There again, indeed, gene alignment is used in various ways to elicit a global collinear response in both time and space.

**Figure 4.**
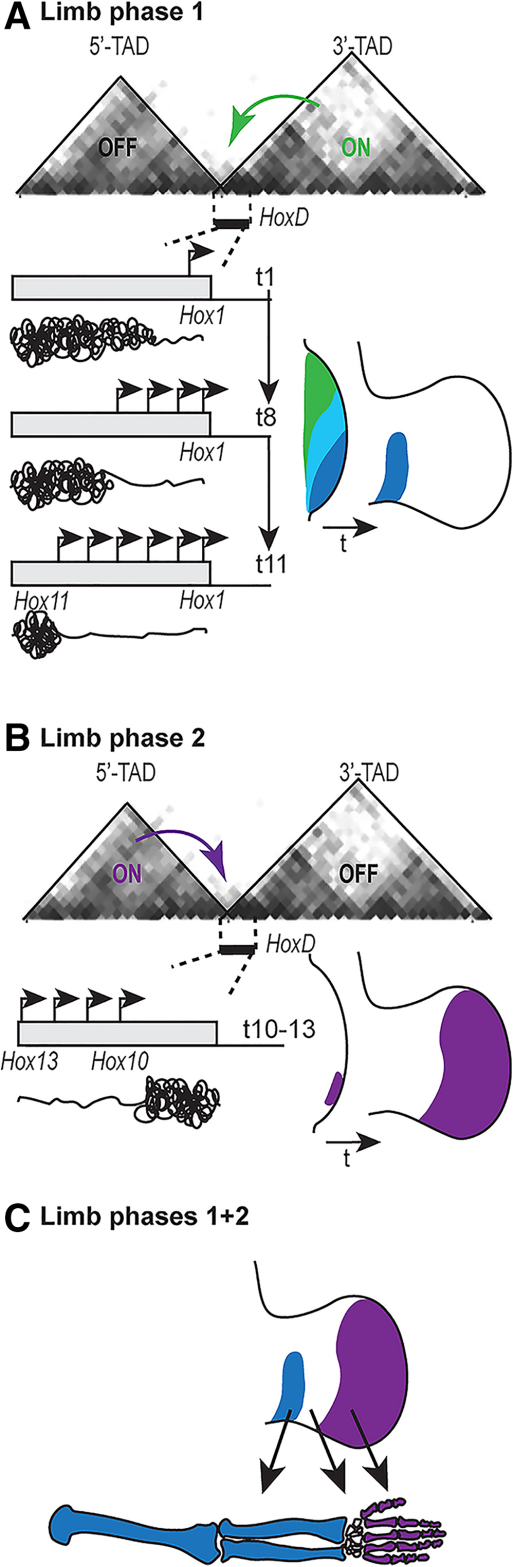
Two temporal sequences in *Hoxd* gene activation during limb bud development. (*A*) Limb phase 1. *HoxD* in the early bud is controlled (green arrow) exclusively by enhancers located in the 3′ TAD. Time sequence of *Hoxd* activation (*t*1 to *t*11) is the same as in the trunk (Fig. 1), and the nested expression domains generated occupy the proximal part of the E12.5 limb bud (right limb bud) and pattern the long bones along with genes from the *HoxA* cluster. Arrows are transcriptionally active genes, corresponding to open chromatin. (*B*) Limb phase 2. Between 24 and 48 h after the start of phase 1, enhancers in the 5′ TAD are switched on and activate *Hoxd10* to *Hoxd13* (purple arrow). HOX13 products then switch off the 3′ TAD enhancers in cells located at the posterior-distal margin. These cells expand and generate the autopods (hands and feet). (*C*) Combination of limb phases 1 and 2. The 3′ and 5′ TADs operate sequentially, leaving a zone of low *Hox* expression as the limb bud grows distally, which generates the future mesopods (wrist and ankle). Anterior is to the top, and distal to the right. The color code for *Hox* gene expression is the same as in Figure 1.

Limb buds arise from the lateral plate mesoderm at defined axial levels. At early stages of limb budding, *Hoxd* genes are transcribed in the same spatio–temporal order as during trunk extension. During this early phase (“limb phase 1”) (Fig. 4), the *Hox* clock is implemented, and 3′-located genes are activated sequentially at the posterior aspect of the limb buds, leading to nested transcript domains, as found with Russian dolls (Fig. 3; Dolle et al. 1989; Nelson et al. 1996; Tarchini and Duboule 2006). During this initial phase of transcription, the underlying mechanism is likely identical to that at work during trunk extension and specifically involves interactions with the 3′ TAD, as shown by targeted chromosome engineering (Andrey et al. 2013). The outcome of this first wave of transcription is to help grow and establish proximal patterns (the arm and the forearm) (Zakany and Duboule 2007 and references therein).

## Transcription timing vs. evolutionary timing

About 24 h after budding, however, cells located at the posterior-distal aspect of the bud start expressing simultaneously a series of “terminal” *Hoxd* genes, including *Hoxd13*. This is the start of limb expression phase 2, which helps pattern the distal part of the limbs (the hands and feet). In these posterior-distal cells, *Hoxd* genes are no longer controlled by the 3′ TAD but instead are controlled by the opposite 5′ TAD (Fig. 3; Montavon et al. 2011; Andrey et al. 2013). At the time the 5′ TAD is switched on, the 3′ TAD enhancers terminate operating such that a second type of temporal collinearity is established, whereby enhancers located 3′ to the *HoxD* cluster work earlier than a large series of 5′-located enhancers. This topological switch in regulations is due in part to a retroactive negative effect of HOX13 proteins upon the activity of the 3′ TAD. This was shown by the genetic removal of the appropriate HOX13 proteins, which prevented the 3′ TAD from switching off; its enhancers continued to operate in most distal limb bud cells, where 5′ TAD enhancers should have normally operated (Beccari et al. 2016; Sheth et al. 2016).

In contrast, the mechanism involved in activating the 5′ TAD remains unknown. Using dissociated limb bud cells cultured in vitro, it was shown recently that the activation of the full set of *Hoxd* genes requires the presence of *Wnt*, *Fgf8*, and *Shh* signaling. In such a system, however, the switch from 3′ to 5′ TAD regulation was not clearly detected (Rodrigues et al. 2017). Notably, this 5′ TAD does not seem to exist in *amphioxus* (see above), raising the possibility of an evolutionary sequence in the appearance of these global regulatory landscapes, with the 5′ TAD emerging subsequently, with the evolution of vertebrates and their appendages (Acemel et al. 2016).

Therefore, during limb development, temporal collinearity comes under two different flavors, which likely reflect distinct key evolutionary steps. Initially, at the time that appendicular skeletons appeared, particular *Hox* combinations were recruited in cells from the lateral plate mesoderm, corresponding to the places of limb bud emergence. These cells had inherited their *Hox* code from their MPs, according to the temporal collinearity rule in the primitive streak. In these cells, the *Hox* clock further processed the state of “cluster opening” that had been memorized in space at these body levels during trunk extension. In this respect, patterning of proximal limb structures shares a genetic “history” with that of the major body axis (Woltering and Duboule 2010).

In a subsequent mechanistic phase, which probably appeared along with the emergence of the autopods (hands and feet), a distinct regulatory topology was recruited to control the last *Hoxd* genes into the newly appearing most distal pieces of our limbs. This second type of temporal collinearity involves 5′ TAD enhancers being switched on after 3′ TAD proximal enhancers and just before the latter regulatory sequences are switched off, thus somewhat recapitulating the evolutionary sequence of events leading to the actual morphology of the tetrapod limb. Interestingly, unlike in the trunk as mentioned previously, the relationship between both phases of *Hox* gene activation and the presence of limb stem cells remains elusive. The existence of such cells throughout limb bud development is controversial, and hence the restricted cell population expressing *Hoxd13* and *Hoxa13* at the start of limb phase 2 may either already have a transitory proximal fate or be kept at an early step of mesoderm commitment (for discussion, see Tabin and Wolpert 2007). In any case, however, these future distal cells went through the activation of the full repertoire of *Hoxd* genes before expanding distally.

## The rationale of temporal collinearity

While other *Hox*-unrelated gene clusters are regulated coordinately (e.g., see Kim and Dean 2012), this precision in meta-*cis* regulation associated with temporal collinearity is thus far unique to *Hox* gene clusters. This time process requires a complete series of genes to be clustered, and hence the physical neighborhood of *Hox* genes at their genomic loci is a fundamental parameter to ensure a faithful anterior-to-posterior patterning in vertebrates. It is likely that this particular process was selected and conserved in vertebrates as well as in all animals developing their body through an anterior-to-posterior time progression (Duboule 2007) because of the double security that it offers in the processing of the patterning information. First, the progressive opening of *Hox* clusters from *Hox1* to *Hox13* following a set of signals and chromatin reorganization allows, in principle, the use of the same few activators for series of genes, the main parameter being promoter accessibility. This may greatly simplify the task, as a precise and sequential activation of series of up to 13 genes within a short time window based solely on a “classical” gene regulatory network in *trans* would have been prone to errors and variations, which cannot be tolerated in the developmental contexts concerned. Likewise, this meta-*cis* organization secures the proper order of the patterning information emitted, since, for example, group 7 *Hox* genes cannot be activated before group 5.

Second, this structural parameter also ensures that the *Hox* genes terminating the process, mostly the members of group 13, are not activated before the whole repertoire of information has been dispatched. Group 13 proteins exert a dominant-negative effect over other HOX proteins through posterior prevalence, leading to the abrupt termination of growth and patterning wherever such proteins are produced, as observed by either spontaneous mutations or induced gain-of-function experiments (e.g., see Herault et al. 1997; Young et al. 2009). Therefore, an essential prerequisite of the system is to prevent *Hox13* genes from being activated before all other genes have achieved their tasks. However, it is equally important that *Hox13* genes are activated as soon as the series has reached its end. In this context, it is likely that the most parsimonious way to properly solve this conundrum was to select such a meta-*cis* regulation, which may explain the evolutionary success of temporal collinearity.
